# Short-term culture for rapid identification by mass spectrometry and automated antimicrobial susceptibility testing from positive bottles

**DOI:** 10.1186/s12879-024-09475-x

**Published:** 2024-06-06

**Authors:** Peng-Peng Tian, Shan-Shan Su, Li-Sha Zhu, Tian Wang, Hui Yang, Meng-Yao Du, Cai-Zhi Ding, Li Wang, Wen Fan, Hua-Wei Yi

**Affiliations:** 1 Laboratory Department, The First Affiliated Hospital of Yangtze University, Jing Zhou, Hubei China; 2 Hubei Provincial Clinical Research Center for Individualized Diagnosis and Treatment of Cancer, The First People’s Hospital of Jingzhou, Jingzhou, Hubei China; 3 Laboratory Department, The People’s Hospital of Songzi, Jingzhou, Hubei China

**Keywords:** Bloodstream infection, Short-term culture, Turn-around time, Rapid identification, Antimicrobial susceptibility testing

## Abstract

**Background:**

Early and appropriate antibiotic treatment improves the clinical outcome of patients with sepsis. There is an urgent need for rapid identification (ID) and antimicrobial susceptibility testing (AST) of bacteria that cause bloodstream infection (BSI). Rapid ID and AST can be achieved by short-term incubation on solid medium of positive blood cultures using MALDI-TOF mass spectrometry (MS) and the BD M50 system. The purpose of this study is to evaluate the performance of rapid method compared to traditional method.

**Methods:**

A total of 124 mono-microbial samples were collected. Positive blood culture samples were short-term incubated on blood agar plates and chocolate agar plates for 5 ∼ 7 h, and the rapid ID and AST were achieved through Zybio EXS2000 MS and BD M50 System, respectively.

**Results:**

Compared with the traditional 24 h culture for ID, this rapid method can shorten the cultivation time to 5 ∼ 7 h. Accurate organism ID was achieved in 90.6% of Gram-positive bacteria (GP), 98.5% of Gram-negative bacteria (GN), and 100% of fungi. The AST resulted in the 98.5% essential agreement (EA) and 97.1% category agreements (CA) in NMIC-413, 99.4% EA and 98.9% CA in PMIC-92, 100% both EA and CA in SMIC-2. Besides, this method can be used for 67.2% (264/393) of culture bottles during routine work. The mean turn-around time (TAT) for obtaining final results by conventional method is approximately 72.6 ± 10.5 h, which is nearly 24 h longer than the rapid method.

**Conclusions:**

The newly described method is expected to provide faster and reliable ID and AST results, making it an important tool for rapid management of blood cultures (BCs). In addition, this rapid method can be used to process most positive blood cultures, enabling patients to receive rapid and effective treatment.

## Introduction

Bloodstream infection (BSI) is one of the leading causes of death worldwide [1, 2]. BSI has caused a significant global healthcare burden, with an estimated mortality rate of 12 ∼ 20% in 2017 [3]. It is well established that the survival of BSI patients depends on the rapid administration of effective antimicrobial therapy [4, 5]. The survival rate decreases by approximately 7.6% when antimicrobial administration is delayed for 1 h [6]. Rapid identification (ID) and antimicrobial susceptibility testing (AST) of the BSI pathogens are essential for timely selection of appropriate antimicrobial therapy, which may result in a better outcome for patients [7, 8]. Blood culture (BC) is the gold standard method for the diagnosis of BSI, which includes processes such as sample collection, incubation, ID, and AST.

The conventional method requires positive BCs to be cultured on solid medium for 18 ∼ 24 h or more before ID and AST. Matrix-assisted laser desorption/ionization time-of-flight mass spectrometry (MALDI-TOF MS) has proven to be a rapid, accurate, and cost-effective technology in the routine identification of microorganisms [9, 10]. The mass spectrometer can provide excellent identification in positive blood cultures and has also been applied in drug resistance, such as direct detection of klebsiella pneumoniae carbapenemase (KPC) based on MALDI-TOF MS [11, 12]. However, these methods require additional labor-intensive, expensive steps in ID, and cannot effectively shorten the time of AST.

In order to reduce the turn-around time (TAT) of current methods, Jihye Ha et al. established a rapid method by short-term incubation of positive blood culture samples on solid culture medium for 6 h followed by ID and AST [13]. Here, this study aims to evaluate the performance of the rapid method. After a 5 ∼ 7 h incubation of positive blood culture samples, MALDI-TOF MS and automated devices were used to detect ID and AST, respectively. In addition, we hope to integrate this method into our routine laboratory to shorten the incubation time for ID and AST, thereby optimizing the standard procedures in microbiology laboratory.

## Methods

### Blood culture samples

Blood culture (BC) bottles (Bactec plus/F; Becton Dickinson, Franklin Lakes, NJ, USA) were collected from patients with suspected BSI between August to September of 2023 at the First Affiliated Hospital of Yangtze University, Jing Zhou, China. The BC bottles were incubated in the Bactec system (Becton Dickinson) until a positive result was obtained or for a maximum of 5 days. In this study, only positive BC samples with Gram staining as distinctly single microorganisms were selected. A total of 124 positive BCs were analyzed using the conventional laboratory diagnostic method and the novel rapid method. If the positive BCs were processed before 10 am, colonies incubated on solid culture medium for about 5 ∼ 7 h could undergo rapid ID and AST around 3: 30 pm in the afternoon.

### Conventional method of ID and AST

After the BD Bactec system displayed a positive signal, Gram staining was performed, and then sub-cultured on blood agar plates (BAP) and chocolate agar plates (CAP) containing vancomycin. These plates were grown in an incubator (Thermo Fisher Scientific, USA) with 5% CO_2_ at 37℃. After overnight incubation, the colonies grown on the BAP were used for ID using MALDI-TOF MS (Zybio EXS2000, China). Bacterial colonies were transferred to the MS target plates using a wooden toothpick. The target plates were overlaid with 1 µL of 70% formic acid. Once the formic acid solution dried, 1 µL of matrix solution was added for subsequent MALDI-TOF MS ID. The calibration and validation of MALDI-TOF MS were carried out once a week with a bacterial test standard according to the manufacturer’s instructions. In brief, a score ≥ 2.0 was interpreted as reliable ID to the species level, a score of 1.7-2.0 was interpreted as reliable ID to the genus level, and a score < 1.7 was interpreted as no reliable ID.

A standardized inoculum (McFarland standard of 0.5) was then prepared from single colonies grown on the BAP, and the appropriate BD Phoenix™ M50 AST panels were chosen according to the ID results provided by Zybio EXS2000 MS. AST plates NMIC-413, PMIC-92, and SMIC/ID-2 were used for Gram negative (GN) bacteria, *Staphylococcus/Enterococcus* spp, and *Streptococci* spp, respectively. AST results were obtained after 18 ∼ 24 h incubation using the BD Phoenix™ M50 instrument and interpreted according to current Clinical and Laboratory Standards Institute. The results of ID and AST obtained using the conventional method were used as the standard for comparison.

### Rapid ID and AST using the short-term incubation method

After 5 ∼ 7 h incubation, microbial ID was obtained directly from the growth of bacteria on BAP by Zybio EXS2000 MS. Colonies on the CAP were taken for AST detection when the rapid ID was shown as GN bacteria. However, for Gram positive (GP) bacteria, the colony on BAP was selected. The suspension obtained from bacterial growth for 5–7 h could be used for AST detection on BD Phoenix™ M50.

The comparisons between rapid and conventional methods were categorized as: category agreement (CA), essential agreement (EA), very major error (VME) (false susceptibility), major error (ME) (false resistance), or minor error (mE) (susceptible/resistant vs. intermediate). In this study, 57 isolates were subjected to both rapid and conventional method for AST detection.

### Evaluation of the turn-around time (TAT) in the conventional method

The turn-around time (TAT) consisted of two components: (i) time to positivity, corresponding to the time required for microorganism growth by Bactec incubation; and (ii) processing time, corresponding to the time required to generate the final report (including bacterial ID, AST, validation of results, and reporting to clinicians).

### Quality control

Standard strains *Staphylococcus aureus* ATCC 29,213 and *Enterococcus faecalis* ATCC 29,212 were used for internal quality control of PMIC-92 plate. *Streptococcus pneumoniae* ATCC 49,619 was used as SMIC/ID-2 QC strains. In addition, standard *Escherichia coli* ATCC 25,922 and *Pseudomonas aeruginosa* ATCC 27,853 were used as internal quality control strains for NMIC-413.

## Results

### Comparison of rapid and conventional ID methods

A total of 124 monomicrobial-positive BCs were collected, including 67 GN bacteria, 53 GP bacteria and 4 *Candida* spp. In addition, 5 samples that were polymicrobial after subculture were excluded. These monomicrobial-positive BCs were detected using the rapid ID method combined with MALDI-TOF MS and compared to conventional method. Among the 67 GN isolates, 61 (91.0%) showed a score higher than 2, while 5 (7.5%) scored between 1.7 and 2, 1 (1.5%) was unidentified (Table 1). Among the 53 GP isolates, 38 (71.7%) demonstrated a score higher than 2, 10 (18.9%) demonstrated a score between 1.7 and 2, and 5 (9.4%) Coagulase-negative staphylococci (CoNS) were incorrectly identified as another CoNS (Table 2). Among 4 *Candida* spp, 3 (75%) demonstrated a score higher than 2, 1 (25%) demonstrated a score between 1.7 and 2 (Table 2).

Compared with the conventional method, the concordance of the rapid ID results of 67 GN bacteria was 98.5% (66/67), while the concordance of 53 GP bacteria was 90.6% (48/53) at the species level, but 98.1% (52/53) at the genus level. The *Candida* isolates exhibited perfect concordance rate of 100% (4/4).

**Table 1:** Gram-negative bacteria from monomicrobial blood cultures identified by rapid and conventional culture-dependent method (*n* = 67)

Organisms	Conventional ID			Rapid ID				
	> 2.0	1.7-2.0	< 1.7	> 2.0	1.7-2.0	< 1.7	un-ID	mis-ID
*E. coli*	39	1		35	5			
*K. pneumoniae*	9			9				
*P. aeruginosa*	3			3				
*C. freundii*	2			2				
*S. marcescens*	2			2				
*E. cloacae*	2			2				
*A. junii*	1			1				
*B. cenocepacia*	1			1				
*A. pitti*	1			1				
*A. radioresistens*	1			1				
*P. mirabilis*	1			1				
*A. veronii*	1			1				
*A. xylosoxidans*	1			1				
*S. typhimurium*	1			1				
*Bru.* spp	1						1	
Total isolates	66	1		61	5		1	

un-ID: unidentified, mis-ID: misidentified

**Table 2:** Gram-positive bacteria and fungi from monomicrobial blood cultures identified by rapid and conventional culture-dependent method (*n* = 57)

Organisms	Conventional ID			Rapid ID				
	> 2.0	1.7-2.0	< 1.7	> 2.0	1.7-2.0	< 1.7	un-ID	mis-ID
*S. hominis*	17	1		11	5			2
*S. epidermidis*	7	1		5	2			1
*S. aureus*	6			6				
*S. capitis*	5			4	1			
*S. haemolyticus*	3			2				1
*S. warneri*	1				1			
*S. lugdunensis*	1	1			1		1	
*E. faecalis*	4			4				
*S. oralis*	1			1				
*S. sanguinis*	1			1				
*S. agalactiae*	1			1				
*S. dysgalactiae*	1			1				
*L. monocytogenes*	1			1				
*C. striatum*	1			1				
*C. glabrata*	2			1	1			
*C. tropicalis*	1			1				
*C. lusitaniae*	1			1				
Total isolates	54	3		41	11		1	4

un-ID: unidentified, mis-ID: misidentified

### Comparison of rapid and conventional AST results using BD M50 AST panels

Among the 124 isolates, 57 isolates were selected for both rapid and conventional AST using BD M50, including 37 *Enterobacteriaceae* (27 *E. coli*, 4 *K. pneumoniae*, 2 *C. freundii*, 2 *E. cloacae*, 1 *S. marcescens*, 1 *S. typhimurium*) and 5 non-fermenting gram-negative rods (1 *P. aeruginosa*, 1 *A. radioresistens*, 1 *A. pitti*, 1 *A. junii*, 1 *A. veronii*), 8 *staphylococcus*, 3 *Enterococcus*, 4 *Streptococcus*. 51.8% (14/27) of *E. coli* and 25.0% (1/4) of *K. pneumoniae* were resistant to Ceftriaxone, 2 isolates were Carbapenem-resistant Enterobacteriaceae (CRE), 3 isolates of *Staphylococci* were methicillin resistant staphylococcus (MRS). For all 42 GN isolates (except *A. junii*), a total of 946 bacterial-antimicrobial combinations were analyzed. Compared to the conventional AST, the EA, CA, mE, ME, VME of the BD M50 NMIC-413 panels by rapid AST were 98.5%, 97.1%, 2.3%, 0.4% and 0.1%, respectively (Table 3). For 8 *Staphylococcus* spp and 3 *Enterococcus* spp, a total of 179 bacterial-antimicrobial combinations were analyzed. The EA, CA, mE of PMIC-92 panels were 99.4%, 98.9% and 1.1%, respectively, while ME and VME were 0% (Table 4). For the SMIC/ID-2 panel, the EA, CA were both 100% of 4 *Streptococcus* spp (Table 5).

**Table 3:** AST Results obtained using the rapid method compared with those of the conventional method in Gram-negative bacteria (41) of NMIC-413

Antimicrobial agent	*N*	*N* (%) of				
		EA	CA	mE	ME	VME
Amikacin	41	41(100)	41(100)			
Gentamicin	41	41(100)	41(100)			
Tobramycin	41	39(95.1)	36(87.8)	4(9.8)	1(2.4)	
Ertapenem	37	37(100)	36(97.3)		1(2.7)	
Imipenem	41	39(95.1)	39(95.1)	2(4.9)		
Meropenem	41	39(95.1)	40(97.6)		1(2.4)	
Cefazolin	37	37(100)	36(97.30)	1(2.7)		
cefuroxime	37	37(100)	37(100)			
cefoxitin	37	37(100)	37(100)			
Ceftazidime	41	41(100)	41(100)			
Ceftriaxone	40	39(97.5)	40(100)			
Cefepime	41	40(97.6)	39(95.1)	2(4.9)		
Aztreonam	39	36(92.3)	36(92.3)	3(7.7)		
Amoxicillin/ clavulanic	37	36(97.3)	36(97.3)			1(2.7)
Ampicillin/sulbactam	39	39(100)	35(89.7)	4(10.3)		
Piperacillin/tazobactam	41	41(100)	39(95.1)	2(4.9)		
Sulfamethoxazole/trimethoprim	40	40(100)	40(100)			
chloramphenicol	40	39(97.5)	38(95)	1(2.5)	1(2.5)	
Ciprofloxacin	41	41(100)	41(100)			
Levofloxacin	41	40(97.6)	40(97.6)	1(2.4)		
Norfloxacin	37	37(100)	37(100)			
minocycline	39	39(100)	38(97.4)	1(2.6)		
tetracycline	40	40(100)	40(100)			
Tigecycline	37	37(100)	36(97.3)	1(2.7)		
Total	946	932(98.5)	919(97.1)	22(2.3)	4(0.4)	1(0.1)

**Table 4:** AST Results obtained using the rapid method compared with those of the conventional method in *Staphylococcus* and *Enterococcus* of PMIC-92

Antimicrobial agent	*N*	*N* (%) of				
		EA	CA	mE	ME	VME
Gentamicin	8	8(100)	8(100)			
ceftaroline	4	4(100)	4(100)			
ampicillin	3	3(100)	3(100)			
penicillin	11	11(100)	11(100)			
Oxacillin	8	8(100)	8(100)			
daptomycin	11	11(100)	11(100)			
Sulfamethoxazole /trimethoprim	8	8(100)	8(100)			
teicoplanin	11	11(100)	11(100)			
Vancomycin	11	11(100)	11(100)			
Clindamycin	8	8(100)	8(100)			
Erythromycin	11	10(90.9)	10(90.9)	1(9.1)		
chloramphenicol	11	11(100)	10(90.9)	1(9.1)		
Linezolid	11	11(100)	11(100)			
Ciprofloxacin	11	11(100)	11(100)			
Levofloxacin	11	11(100)	11(100)			
Rifampin	8	8(100)	8(100)			
minocycline	11	11(100)	11(100)			
tetracycline	11	11(100)	11(100)			
Tigecycline	11	11(100)	11(100)			
Total	179	178(99.4)	177(98.9)	2(1.1)		

**Table 5:** AST Results obtained using the rapid method compared with those of the conventional method in *Streptococci* of SMIC/ID-2

Antimicrobial agent	*N*	*N* (%) of				
		EA	CA	mE	ME	VME
Meropenem	4	4 (100)	4 (100)			
cefotaxime	4	4 (100)	4 (100)			
cefepime	4	4 (100)	4 (100)			
amoxicillin	4	4 (100)	4 (100)			
penicillin	4	4 (100)	4 (100)			
Vancomycin	4	4 (100)	4 (100)			
Clindamycin	4	4 (100)	4 (100)			
Erythromycin	4	4 (100)	4 (100)			
chloramphenicol	4	4 (100)	4 (100)			
Linezolid	4	4 (100)	4 (100)			
Levofloxacin	4	4 (100)	4 (100)			
tetracycline	4	4 (100)	4 (100)			
Total	48	48 (100)	48 (100)	0	0	0

### Evaluation of the TAT

During the study, we collected a total of 393 positive BC bottles, of which 264 (67.2%) were able to utilize the rapid ID and AST method. The mean TAT for detecting and reporting 57 isolates using conventional and rapid method were 73.0 ± 10.7 h and 48.9 ± 10.2 h, respectively. The TAT of the rapid method was shortened by about 24 h (Table 6).

**Table 6:** Mean and standard deviation value of times in conventional method

Times (h)	Mean	GN	GP
Time to positivity	11.7 ± 4.8 (3.3 ∼ 24.7)	10.7 ± 4.2 (3.3 ∼ 18.7)	14.6 ± 5.2 (7.7 ∼ 24.7)
*Conventional method*			
Processing time	61.3 ± 12.4 (27.4 ∼ 87.6)	62.4 ± 11.8 (35.6 ∼ 87.6)	57.9 ± 13.9 (27.4 ∼ 79.5)
TAT	73.0 ± 10.7 (48.1 ∼ 96.1)	73.1 ± 10.6 (48.3 ∼ 96.1)	72.5 ± 11.5 (48.1 ∼ 90.2)
*Rapid method*			
Processing time	37.2 ± 11.8 (14.1 ∼ 65.2)	38.3 ± 11.7 (14.1 ∼ 65.2)	34.2 ± 12.2 (16.0 ∼ 57.1)
TAT	48.9 ± 10.2 (26.7 ∼ 73.7)	49.0 ± 10.4 (26.7 ∼ 73.7)	48.8 ± 9.8 (36.7 ∼ 67.7)

Minimum and maximum values are reported in brackets

## Discussion

Immediate administration of appropriate antibiotics is necessary for the effective treatment of bacteremia, as any delay is associated with increased morbidity and mortality [14]. MALDI-TOF MS has been proven to directly identify bacteria in positive BCs [2, 15, 16]. However, it has drawbacks such as time-consuming, complicated operation, and expensive [17, 18]. Molecular methods have shown to be efficient for the rapid ID of specific microorganisms but can identify a limited range of microorganisms and antimicrobial resistance genes. Nucleic acids-based technologies could be used for sensitive detection of bloodstream pathogens directly from a blood sample. Due to target limitations, most commercial platforms require a combination of standard blood cultures and adjunctive molecular detection. Additionally, all methods still require a culture step to obtain isolates for comprehensive AST [19]. Rapid AST methods have been developed in recent years, including EUCAST rapid AST and CLSI disk diffusion using positive blood culture medium [20–21]. However, the rapid AST methods require strain identification and cannot be performed in laboratories without mass spectrometry instruments. Moreover, their manual setup and the imperative requirement to read the inhibition zone diameters at strictly defined time points are tremendously labor-intensive [20]. Although the rapid AST has important value for some critically ill patients, its shortcomings hinder its large-scale use in clinical microbiology laboratories. In addition, the CLSI method detects a limited variety of bacterial strains, which cannot meet clinical needs.

The application of MALDI-TOF MS for identifying bacterial colonies from solid media has significantly improved and accelerated routine microbiological diagnostics [8, 22]. Some studies have described a new process based on the short-term incubation method for identifying bacterial pathogens, including ID and AST, from blood cultures [13, 23, 24]. One study showed that the optimal incubation time to ascertain GP bacterial ID was 4.5 h, but for GN bacterial ID was 3.5 h, the identification rates were 97.4% for GN bacteria and 100% for GP bacteria when compared to the conventional method [23]. Another study showed that the species-level ID concordance rate after 6 h of incubation was 90.9% (159/175), and 80.6% (141/175) after 4 h of incubation [25]. To facilitate operation and standardization, we used the incubation time of 5 ∼ 7 h for GP, GN bacteria and fungi. In this study, 9 bacterial strains were incubated for 5 h, 11 strains were incubated for 6 h, and 37 strains for 7 h, with an average incubation time of 6.5 h (median time of 7 h). There was no significant difference in the consistency of ID and AST between strains cultured for 5–7 h. Then, the rapid ID and AST of colonies were done by Zybio EXS2000 MS and BD M50, respectively. This means that processing positive BC samples, as well as ID and AST, can be completed on the same day.

With respect to rapid ID, our results demonstrate that the performance of the presented method is very high and satisfactory for both GP and GN isolates. In our study, only 1 GN bacteria was unidentified, it turned out to be *Brucella* spp with a time to positive of nearly 4 days. The cause of this discrepancy might be the very slow growth of *Brucella*, which could result in a failure to produce sufficient proteins for accurate MALDI-TOF MS analysis. Besides, 1 strain of *Enterobacter cloacae* was misidentified as *Enterobacter asburiae*. Since both belong to the *Enterobacter cloacae complex*, we consider that the ID results are consistent. Low identification scores (< 2.0) were mainly associated with CoNS such as *Staphylococcus epidermidis*, *Staphylococcus hominis*, and *Staphylococcus capitis*. A total of 5 GP isolates were incorrectly identified, including 1 strain of *Staphylococcus lugdunensis* was unidentified, and 4 CoNS strains were misidentified (including 2 *S. hominis*, 1 *S. haemolyticus* and 1 *S. epidermidis*), but they were consistent at genus level. No CoNS were mis-identified as *S. lugdunensis*, which is virulent and the symptoms are similar to that of *S. aureus*. However, CoNS were usually recognized as contaminant species in blood samples, our method showed a greater accuracy when excluding these contaminant strains. The concordance rate of fungi ID was 100%. Moreover, previous studies have shown that the ID rate of GN bacteria is higher than that of GP bacteria [26, 27], and our study has reached similar conclusions.

Regarding the applications of this rapid procedure for AST, positive BCs for GN bacteria were assessed with 24 antimicrobial agents using NMIC-413. The rates of EA, CA, mE, ME, VME were 98.5%, 97.1%, 2.3%, 0.4% and 0.1%, respectively. Meanwhile, 11 isolates of GP were evaluated for 19 antimicrobial agents using PMIC-92, the rates of EA, CA, mE were 99.4%, 98.9% and 1.1%. Similar findings have been reported in previous studies [13, 24]. It is noteworthy that 4 isolates of *Streptococci* were assessed for 12 antimicrobial agents using SMIC/ID-2, both EA and CA exhibited flawless outcomes with a rate reaching 100%. The rapid AST showed excellent results with low error percentage (mE, ME, VME) meeting the performance standards of the U.S. Food and Drug Administration (FDA) (mE < 3%, VME < 1.5%) [28]. It should be noted that colonies on the CAP were used for rapid AST in GN bacteria to avoid contamination from other GP bacteria. One *Acinetobacter junii* was excluded due to the AST without acquired MIC in both rapid and conventional methods, and finally results of AST were obtained by KB method. ME and VME were found only in 3 isolates (1 *Escherichia coli*, 1 *Enterobacter cloacae*, 1 *Salmonella typhimurium*). On the other hand, mE was mostly observed with Ampicillin/sulbactam, Tobramycin, Aztreonam. In the isolated *Staphylococci*, 100% EA and CA were detected for oxacillin, indicating that our method is suitable for detecting methicillin-resistant Staphylococcus (MRS). Finally, in addition to high performance rates for ID and AST, the rapid method greatly shortens the processing time, resulting in the reporting time being about 24 h earlier than traditional methods (shortening TAT by nearly one-third). The results of rapid method have been recognized by many clinicians in our hospital.

The rapid method used in this study also has some limitations. The method cannot be used for polymicrobial-positive BCs. Approximately 3.9% (5/129) of the positive BCs collected in this study were detected as polymicrobial, which is slightly lower than other studies [24, 25]. Besides, the commercialized antimicrobial susceptibility testing panel used in our laboratory do not contain novel β-lactam combination agents, such as imipenem-relebactam, meropenem-vaborbactam, Ceftazidime-avibactam, and no AST was performed on these new drugs in this study. However, there are several highlights in our article. Firstly, the short-term culture 5 ∼ 7 h on solid medium method for positive BCs showed a high concordance (> 90%). Secondly, the rapid method exhibited excellent performance in AST using different AST panels of BD-M50 system. Thirdly, this new procedure allows for a reduction in TAT by nearly 24 h. In summary, the rapid method can be applied to most positive BC samples and provides a workflow friendly approach that can obtain results faster and more reliably, which is very beneficial for the treatment of sepsis patients.

## Data Availability

The datasets used and analyzed during the current study are available from the corresponding author on reasonable request.
